# Transition-to-residency: pilot innovative, online case-based curriculum for medical students preparing for pediatric internships

**DOI:** 10.1080/10872981.2021.1892569

**Published:** 2021-02-23

**Authors:** Marguerite Costich, Morgan A. Finkel, Suzanne Friedman, Marina Catallozzi, Rachel J. Gordon

**Affiliations:** a Department of Pediatrics, Columbia University Medical Center, New York, NY, USA; b Departments of Pediatrics and Population and Family Health, Columbia University Medical Center, New York, NY, USA; c Departments Medicine and Epidemiology, Columbia University Medical Center, New York, NY, USA

**Keywords:** Hybrid learning, transition-to-residency, undergraduate medical education

## Abstract

**Background**: There is increasing recognition in medical education that greater emphasis must be placed on preparing graduating medical students for their new roles as interns. Few publications in the literature have described transition-to-residency curricula specifically for students interested in pediatrics or pediatric-related fields

**Approach**: We developed novel online pediatric cases, embedded within an innovative, hybrid transition-to-residency course, to address high yield, multi-disciplinary topics within the context of several of the AAMC’s identified Entrustable Professional Activities

**Evaluation**: The pilot cases were evaluated over two academic years (2018, 2019) at a single academic medical center as part of routine student course evaluation (N = 18/35) with the 2019 evaluation containing additional retrospective pre-post survey questions (N = 9/18) assessing self-reported changes in knowledge. Almost all students were very satisfied or satisfied with the modules overall (94%), the quality of the resources provided (100%), and the structure and clarity of the presentation of the material (100%). Among the students who completed the retrospective pre-post survey after participation in the online modules, significant self-reported improvements were noted in writing orders to the pediatrics floor (Z = −2.07, p = 0.04), providing anticipatory guidance (Z = −2.0,p = 0.046), formulating a differential diagnosis for common pediatric conditions (Z = −2.24, P = 0.03), and preparedness for managing common pediatric floor emergencies (Z = −2.33, P = 0.02).

**Reflection**: We demonstrated feasibility of implementation of an interactive, online case-based curriculum, medical student satisfaction with content and delivery, and increased self-reported knowledge after completion of the pilot pediatric cases on the online, asynchronous learning platform.

## Background and need for innovation

Literature addressing transition-to-residency courses is expanding as recognition of the need to better prepare students for their roles as interns grows [1–3]. In 2014, the American Association for Medical Colleges (AAMC) published new guidelines that detailed 13 Entrustable Professional Activities (EPAs) that all medical students should be able to perform upon entering residency as a response to evidence that medical school students are sub-optimally prepared for residency[4]. Few publications have described transition-to-residency curricula specifically for students starting pediatric training[5]. While more recent literature has described the movement of such curricula online via applications such as Zoom, the structure of curricula remains essentially the same, with learning occurring synchronously, but at a distance[6]. To our knowledge, there is no literature describing the use of an interactive online, asynchronous platform for delivery of such transition-to-residency curricula. Recent literature has demonstrated that adult learners prefer asynchronous models to traditional didactics, with greater learner participation and enhanced retention of knowledge [7–10]. Despite this, there has not yet been a significant uptake in online, asynchronous learning platforms in transition-to-residency curricula.

Ready 4 Residency (R4R) is an innovative hybrid course created in 2015, with learning occurring online in interactive, asynchronous management-based cases, and in the classroom using case-based sessions and simulation exercises[11]. It is a month-long, mandatory course offered to fourth year students in March or April of their graduating year. The course is run over two sessions, with approximately half of the graduating class participating in each session, or about 60 students. Students attend two or three interactive didactic sessions per day, with time built into the schedule to allow for simulation-based learning, independent learning, and participation in the online component of the course. The pilot pediatric R4R online case was developed within the existing R4R curricula in the spring of 2018 to meet student demand, as the online component of the course previously had no pediatric offerings. A second pediatrics case was introduced in the spring of 2019.

## Goal of innovation

The objective of these cases was to engage fourth year medical students training in pediatrics in patient care scenarios frequently seen in intern year. The interactive cases were designed in such a way as to also address several of the EPAs described by the AAMC (Table 1). Student course evaluations aimed to demonstrate feasibility of implementation of the pilot online asynchronous pediatric cases, evaluate satisfaction with content and delivery, and assess for changes in self-reported knowledge via a retrospective pre-post survey with Likert scale-type questions.

**Table 1.** Examples of learning objectives from case 1

Learning Objective	EPA
Formulate differential diagnosis for respiratory distress in the newborn	EPA 2: Prioritizing a differential diagnosis following a clinical encounter
Use the I-PASS mnemonic to give patient sign-out on admitted patient	EPA 8: Give or receive a patient handover to transition care responsibility
Write admission orders for an infant admitted with acute respiratory distress	EPA 4: Enter and discuss orders and prescriptions
Manage acute respiratory distress on the inpatient unit	EPA 10: Recognize a patient requiring urgent or emergent care and initiate evaluation and management
Interpret commonly used diagnostic tests (e.g., reading chest radiographs, common laboratory tests etc.)	EPA 3: Recommend and interpret common diagnostic and screening tests

## Steps taken for development and implementation of innovation

The cases were designed using a case-based constructivist conceptual framework[12]. The first case provides an overview of clinical scenarios including newborn care, respiratory distress, and fever in a neonate, commonly encountered as interns and follows a infant from initial visit in the ambulatory care setting through the emergency department visit for evaluation and management of bronchiolitis and then ultimately the inpatient general pediatrics unit where the infant is worked up for fever. The second case follows a child with a limp from the outpatient setting through inpatient admission to the floor, and ultimately, the pediatric intensive care unit, providing exposure to multiple clinical settings and covering topics that include septic arthritis and septic shock. The topics reviewed in the cases were selected because they were identified by faculty and residents to be high-yield and involving multiple learning points, disciplines and subspecialties. Among faculty who reviewed the cases were general pediatricians in ambulatory care, and subspecialists in emergency medicine, hospitalist medicine, and pediatric intensive care. The American Academy of Pediatric guidelines, as well as recent evidence in the literature pertaining to each specific learning objective, provided the foundation for the cases[13]. The EPAs highlighted during the cases (i.e., developing a prioritized differential, giving or receiving a patient handover, forming clinical questions and receiving high-quality evidence to advance patient care) have been highlighted in the literature as having the largest gaps between expected and observed performance[14].

The cases were accessible through Courseworks2 (Canvas), an online, interactive learning management system (LMS). Participation in both pediatric R4R cases was elective and occurred during a designated week. The interactive cases were designed to be asynchronous, and students could complete them at their own pace during that week. Students proceeded through the cases independently but were assigned to teams to promote group discussion during designated parts of the case. The teams were comprised of five to six students. The students proceeded sequentially though the case using multiple interactive elements available via the LMS to evaluate, diagnose and manage their patients. Content focused multiple choice ‘Attending Questions’ were drawn from the articles or guidelines the students were asked to review as they worked through the cases.

As the students progressed in the case, they completed assignments, and received feedback from facilitators related to written orders, discharge instructions and identification of applicable evidence and articles.

‘Rounds’ were conducted via a discussion forum where students provided and discussed free text responses with teammates and facilitators. Discussion ‘rounds’ included, but were not limited to, review of laboratory values and radiographs, hand-offs, and development of differential diagnoses, assessments, and plans. Discussion ‘rounds’ were not conducted in real-time but rather through messaging and posting through the LMS.

Students were required to answer each multiple-choice question and discussion ‘rounds’ as the case would not advance unless an answer was provided. Answers provided did not have to be correct to proceed and responses were not graded. Rather, students received points for participation in each interactive activity, with the winning team earning a prize at the completion of the cases.

Facilitators provided individualized feedback and moderated dialogue among students, promoting active learning and independent inquiry. Facilitators, which included faculty, pediatric fellows, and residents, all received training in the month prior to the launch of the cases based on best practices regarding training facilitators for asynchronous discussion [15,16]. They all received an instructive video regarding use of the interactive LMS platform and handouts providing additional guidance on how to provide tailored feedback on an online forum to promote critical thinking and reasoning. Facilitators also received facilitator guides for each of the two cases that featured suggested talking points to further guide discussion and lend uniformity to responses to student remarks. This faculty development training was important, as few of the facilitators had participated in teaching on an asynchronous learning platform prior to this experience. Facilitators were instructed to check back to their assigned portion of the cases at least 2–3 times per day for the duration of the 1-week case and respond to every student comment. Self-report was used to monitor facilitator contribution time from individual facilitators, with curriculum leaders monitoring that all comments were received responses. Students were encouraged to revisit the discussion boards daily to review feedback and other students’ responses. Several of the faculty facilitators were teaching faculty in the course. Others faculty facilitators were interested volunteers, all of whom already had significant interaction with students during their clerkship or sub-internship rotations. Resident facilitators were participating on the ‘Resident as Teacher’ rotation and engagement in the R4R online cases was part of the rotation requirements. Approximately 4–8 facilitators participated in moderating each online case. Each facilitator was assigned one ‘clinical day’ in the case (for example, ‘Day 2-ED’), and ideally, each ‘clinical day’ would be assigned to two facilitators. Facilitators would moderate discussion boards for 1–2 teams, each consisting of 5–6 students.

Anonymous online student course evaluations were administered in 2018 and then again with additional questions in 2019 via Qualtrics and were reviewed by the Center for Education Research and Evaluation at Columbia prior to distribution. The evaluation included eight questions that utilized a 4-point Likert type scale (1 = Very Satisfied, 4 = Very dissatisfied,) that assessed satisfaction and perceptions of the case, as well as 3 open-ended questions assessing feedback on the case. Data from the 2018 and 2019 evaluations were combined for this portion of the analysis. The 2019 evaluation included 16 additional retrospective pre-post- questions that assessed student knowledge level on a 5-point Likert scale (1 = Strongly Agree, 5 = Strongly Disagree) with tasks expected of pediatric interns. Recent literature has demonstrated that retrospective pre-post surveys are as effective for program evaluation purposes as traditional pre-post surveys [17,18]. Additionally, because the questions were administered as part of the routine course evaluation, it was not feasible to administer a traditional pre-survey. The Wilcoxon signed rank test was used to compare the responses to questions about knowledge prior to and post the modules. IBS SPSS Statistics Version 25 was used for all analyses. The open-ended responses were assessed for general themes. The study was exempt by the Institutional Review Board. Students were not consented to participate as the data was collected as part of routine course evaluation. Completion of the online pediatric modules and evaluation was also optional. The evaluations were administered to students immediately after the completion of the online modules.

## Evaluation of innovation

Overall, 35 students (15 in 2018 and 20 in 2019) completed the cases. Nine students each year filled out the post-case survey, resulting in 60% and 45% response rates, respectively (Table 2). Most students in both years were very satisfied or satisfied with the modules overall (94%, n = 17) and felt that the online facilitators were very knowledgeable or knowledgeable (100%, n = 18). Students were very satisfied or satisfied with the quality of the resources provided (100%, n = 18) and structure and clarity of the presentation of the material (100%, n = 18). Most students reported that they worked on the module 1–2 hours per day (61%, n = 11).

**Table 2.** Student satisfaction responses (n = 18)

Questionnaire Prompts	Responses	n (%)
Please rate the difficulty level of the pediatric modules.	Not difficult Somewhat difficult Difficult	6 (33) 11 (61) 1 (6)
Approximately how many hours did you spend each day on the module?	Less than 1 hour 1–2 hours More than 2 hours	5 (28) 11 (61) 2 (11)
How knowledgeable were the online moderators who participated in the modules?	Knowledgeable Very knowledgeable	4 (22) 14 (78)
Please rate your satisfaction with the quality of the resources included in the modules.	Satisfied Very Satisfied	9 (50) 9 (50)
Please rate your satisfaction with the structure and clarity of the presentation of the material.	Satisfied Very Satisfied	8 (44) 10 (56)
Overall, please rate your satisfaction with the pediatric R4R modules.	Dissatisfied Satisfied Very Satisfied	1 (6) 6 (33) 11 (61)

Among the 9 students who completed the post-case evaluation in 2019, significant self-reported improvements after the module were noted in knowledge about writing admission orders to the pediatrics floor (Z = −2.07, p = 0.04), providing anticipatory guidance (Z = 2.0, p = 0.046), formulating a differential diagnosis for common pediatric conditions (Z = −2.24, p = 0.03), and preparedness for managing common pediatric floor emergencies (Z = −2.33, p = 0.02) (Table 3).

**Table 3.** Comparison of prior and post-module responses about clinical knowledge (n = 9)

Questionnaire Prompt*	Prior Median (IQR)	Post Median (IQR)	Z^‡^	p
I know how to interpret commonly used diagnostic tests (e.g., reading chest radiographs, reviewing laboratory tests).	2 (1–2)	1 (1–2)	−1.73	0.08
I know how to write admission orders to the pediatric floor.	2 (1.5–3.5)	1 (1–2)	−2.07	0.04^†^
I know how to provide anticipatory guidance to families in multiple settings for common issues such as when to seek medical care.	2 (2–2.5)	2 (1–2)	−2.0	0.046^†^
I know how to formulate a differential diagnosis for common pediatric conditions such as respiratory distress and refusal to bear weight.	2 (1–2)	1 (1–1)	−2.24	0.03^†^
I know how to conduct an evidence-based medicine literature search to obtain an answer to a clinical question.	1 (1–1.5)	1 (1–1.5)	0.0	1.00
I feel prepared to manage common pediatric floor emergencies such as respiratory distress.	2 (2–2)	1 (1–2)	−2.33	0.02^†^
I know how to sign out patients using a one-liner statement and the IPASS pneumonic.	2 (1.5–2)	1 (1–2)	−1.73	0.08

*1 = Strongly Agree, 2 = Somewhat Agree, 3 = Neither Agree nor Disagree, 4 = Somewhat disagree **^‡^**Wilcoxon Signed Ranks Tests

^†^p < 0.05

Written feedback reflected an overall positive response to learning opportunities highlighted in the interactive cases. In response to the prompt asking what they liked most about the cases, the most frequently mentioned components were moderator feedback and comments (n = 6), articles and guidelines (n = 5), high yield cases (n = 5), and practicing intern specific tasks like discharge summaries and orders (n = 3). The area of improvement suggested most frequently was a desire for a more challenging and complex case (n = 5).Informal feedback collected from facilitators demonstrated ease of use of the online, asynchronous model following the faculty development training. Faculty members reported spending between 30 and 60 minutes a day responding to student comments.

## Critical reflection

We have demonstrated that the implementation of innovative, online interactive pediatric cases for graduating fourth year medical students was well-received. Students were satisfied with the content and delivery of the curriculum and findings from the retrospective pre-post evaluation demonstrate that there was self-reported improvement in knowledge in numerous topic areas addressed by the curriculum.

The literature describes several introduction to residency courses that have been implemented in past years in medical schools [2,19]. However, to date, we are not aware of any programs specifically geared toward students training in pediatrics and the unique skills necessary for the field. In addition, the role of hybrid and asynchronous learning has been demonstrated but this is particularly important now given recent changes in the medical education landscape where social-distancing guidelines have necessitated a transition to online learning[20]. As the pediatric R4R curriculum is asynchronous and able to be flexibly implemented, similar modules may be employed in other clinical settings, owing to ease of scheduling and need for limited in-person faculty involvement.

We have also demonstrated that faculty development can help facilitate use of asynchronous learning modalities for all levels of educators and promote engagement with learners on online platforms.

The use of facilitators provides students with the advantage of individualized feedback in a case-based scenario. This differentiates the R4R pediatric cases from completely asynchronous cases which do not allow the students the ability to interact and receive tailored feedback from local faculty and resident and fellow trainees[21].

With regard to resident and fellow facilitators, evidence suggests that near peer-assisted learning among undergraduate medical students is well received and a useful mechanism for providing formative feedback[22]. The online discussion forums also provide students with the opportunity to learn from each other’s responses, contributing to peer learning[23].

EPAs provide the structural framework for the asynchronous, online cases, allowing for generalizability beyond pediatrics. While the specific knowledge content will vary based on the field of medicine of the learner, the task-oriented objectives transcend specialty fields as fulfillment of EPAs is widely accepted as a prerequisite to starting residency[24].

Our project has several limitations. A formal needs assessment was not completed prior to case design. This is a limitation as key-stakeholders, namely recently medical school graduates in pediatrics, were not surveyed. Additionally, these were pilot cases, and few medical students have completed the cases to date. Completion of the cases was also optional, and surveys were obtained only from the students who completed the cases as part of routine course evaluation. Total survey participation was low adding an additional limitation. Those who completed the cases were also a self-selecting group, interested in pediatrics, and results of the surveys may be reflective of this. The surveys, therefore, lack an appropriate control group, and we are unable to assess whether increased knowledge in performing the tasks described in the survey can be attributed to the cases alone or whether as a result of increased experience overtime.

Whereas the 2018 evaluation only evaluated reaction, the 2019 evaluation was expanded to assess impact of the curriculum on knowledge acquired[25]. However, such questions were assessed retrospectively, and potentially affected by recall bias. Of note, students’ self-perception of knowledge was high even prior to completion of the modules, and while there was a statistically significant improvement in self-perception of knowledge, the difference was less than one point on all measures. Nonetheless, while small, these improvements are still important as they relate to key topic areas such as provision of anticipatory guidance where even small differences can lead to substantial reduction in potential for patient harm.

Because the curriculum is new, we also do not know whether these cases will have an impact on medical care, though findings from our preliminary analysis suggest that students feel more skilled with their clinical decision-making following the curriculum.

Future steps may include a longitudinal survey distributed to interns who completed R4R to assess whether the knowledge obtained from participation in the pediatric cases had any meaningful impact on preparedness for intern year. Feedback from interns may also be used to suggest additional content areas for current and future cases. Similarly, analysis of intern service exam scores would similarly be helpful for identifying areas for improvement. We also plan to obtain qualitative feedback from faculty, fellow, and resident facilitators regarding their experiences participating in this asynchronous curriculum. There is a body of literature to suggest that faculty often do not engage in asynchronous, hybrid learning teaching practices because it is felt that they do not have adequate training or time [26,27]. We would like to know more about our facilitators’ experiences participating in the curriculum to facilitate further faculty development for future asynchronous learning curriculums.

While further evaluation is needed, given the content and delivery, these interactive cases may be considered for use as a tool to prepare medical students prior to graduation of medical school and start of residency.
